# Bone Morphogenetic Protein-9 Is a Potent Chondrogenic and Morphogenic Factor for Articular Cartilage Chondroprogenitors

**DOI:** 10.1089/scd.2019.0209

**Published:** 2020-07-09

**Authors:** Ben J. Morgan, Guillermo Bauza-Mayol, Oliver F. W. Gardner, Yadan Zhang, Riccardo Levato, Charles W. Archer, Rene van Weeren, Jos Malda, Robert Steven Conlan, Lewis W. Francis, Ilyas M. Khan

**Affiliations:** ^1^ Centre of Nanohealth, Swansea University Medical School, Swansea, United Kingdom.; ^2^ Stem Cells and Regenerative Medicine, UCL Great Ormond Street Institute of Child Health, London, United Kingdom.; ^3^ Department of Orthopaedics, University Medical Center Utrecht, Utrecht, the Netherlands.; ^4^ Department of Equine Sciences, Faculty of Veterinary Medicine, Utrecht University, Utrecht, the Netherlands.

**Keywords:** chondroprogenitors, BMP9, GDF2, anisotropic, differentiation, cartilage

## Abstract

Articular cartilage contains a subpopulation of tissue-specific progenitors that are an ideal cell type for cell therapies and generating neocartilage for tissue engineering applications. However, it is unclear whether the standard chondrogenic medium using transforming growth factor beta (TGFβ) isoforms is optimal to differentiate these cells. We therefore used pellet culture to screen progenitors from immature bovine articular cartilage with a number of chondrogenic factors and discovered that bone morphogenetic protein-9 (BMP9) precociously induces their differentiation. This difference was apparent with toluidine blue staining and confirmed by biochemical and transcriptional analyses with BMP9-treated progenitors exhibiting 11-fold and 5-fold greater aggrecan and collagen type II (COL2A1) gene expression than TGFβ1-treated progenitors. Quantitative gene expression analysis over 14 days highlighted the rapid and phased nature of BMP9-induced chondrogenesis with sequential activation of aggrecan then collagen type II, and negligible collagen type X gene expression. The extracellular matrix of TGFβ1-treated progenitors analyzed using atomic force microscopy was fibrillar and stiff whist BMP9-induced matrix of cells more compliant and correspondingly less fibrillar. Polarized light microscopy revealed an annular pattern of collagen fibril deposition typified by TGFβ1-treated pellets, whereas BMP9-treated pellets displayed a birefringence pattern that was more anisotropic. Remarkably, differentiated immature chondrocytes incubated as high-density cultures in vitro with BMP9 generated a pronounced anisotropic organization of collagen fibrils indistinguishable from mature adult articular cartilage, with cells in deeper zones arranged in columnar manner. This contrasted with cells grown with TGFβ1, where a concentric pattern of collagen fibrils was visualized within tissue pellets. In summary, BMP9 is a potent chondrogenic factor for articular cartilage progenitors and is also capable of inducing morphogenesis of adult-like cartilage, a highly desirable attribute for in vitro tissue-engineered cartilage.

## Introduction

Stem cell therapies have been used in numerous studies in attempts to repair cartilage lesions, but thus far, no solution has been able to regenerate the full adult structure of this tissue [1]. The most obvious route to successful cell therapies lies in understanding and mimicking endogenous growth and developmental processes, either to grow cartilage in vitro or endow implanted cells with sufficient cues to differentiate and develop functional repair tissue. Central to achieve these objectives is the use of progenitor cells receptive to specific instructive cues as well as the use of well-defined differentiation factors to drive the generation of neocartilage [2,3].

Cartilage lesions are a common occurrence during exploratory knee arthroscopic surgery with a reported prevalence of 65% [4]. If these lesions are not repaired they may over time progress to diffuse osteoarthritis [5]. Their treatment remains difficult because intrinsic repair mechanisms are inadequate mainly due to the avascular nature of cartilage tissue and the inability of resident chondrocytes to mobilize and direct repair [6,7]. Cell transplantation and subchondral bone stimulation [8] have been developed to repair localized lesions [9], but crucially neither technique can significantly reduce the medium and long-term risk of patients developing osteoarthritis [10,11]. In a 15-year follow-up study, Knutsen et al. found that 57% of patients treated with cell transplantation and 48% of patients treated by microfracture had radiographic signs of early osteoarthritis [11].

In contrast, osteochondral allografting is a highly effective surgical technique to repair large >2 cm^2^ cartilage defects in synovial joints [12,13]. Cartilage and adjoining bone from donors are transplanted to areas in recipients requiring resurfacing with the aim of restoring normal joint function. The principal advantages of allotransplantation are the use of structurally intact hyaline cartilage and bone with no concurrent morbidity as would be the case for autografting. Clinical studies using cartilage allografts have showed favorable outcomes, with graft survival as high as 95% at 5 years and 85% at 10 years [14]. The limitations of allografting are the availability and storage of donor cartilage, with storage beyond 21 days causing a significant reduction in cellular viability that impact graft quality and survival [15]. Tissue engineering has the potential to fulfill the increasing demand for allografts through the use of expandable cell sources capable of maintaining chondrogenic potency to generate functional hyaline neocartilage.

Donor chondrocytes from recipients cannot be used to repair larger cartilage defects because they undergo dedifferentiation and progressively lose the capacity to redifferentiate after 4–5 population doublings [16]. Consequently, the size of cartilage lesions that can be repaired, typically requiring concentrations of several million cells per cm^2^, is also limited [17]. To overcome this hurdle, adult mesenchymal stem cells (MSCs) such as bone marrow-derived stromal cells with the capacity to undergo chondrogenic differentiation have been used for cell therapy either by autologous implantation into defects [18] or through marrow stimulation through drilling the subchondral bone plate [8]. Marrow-derived MSCs generate a transient fibrocartilagenous template with biomechanical deficits that compromise cell survival in the adult joint [19]. More detailed examination of marrow-derived MSC function suggests that their therapeutic effects are mediated through paracrine effects on recruited cells or coimplanted chondrocytes [20,21]. Therefore, the discovery of tissue-specific progenitor-like cells in articular cartilage with the ability to generate the cell numbers required for cell therapies and tissue engineering is of great significance [22,23].

Articular cartilage-derived chondroprogenitors were first detected as bromodeoxyuridine label retaining cells in the superficial zone of fetal marsupial articular cartilage [23,24]. Subsequently, cells with colony-forming ability were isolated from postnatal immature bovine cartilage that fulfilled the minimal requirements to be classified as mesenchymal progenitors; exhibiting plastic adherence, CD90^+^, CD105^+^, CD73^+^, CD166^+^, CD34^−^, and CD45^−^ antibody reactivity, multipotential phenotypic plasticity, and the ability to home joint tissues [22,25]. Articular cartilage-derived chondroprogenitors maintain sex determining region Y-box 9 (SOX9) expression and telomere length following extensive culture expansion, accounting for their ability to undergo chondrogenic redifferentiation following more than 30 population doublings [26]. Critically, studies have found runt-related transcription factor-2 (RUNX2) and collagen type X, markers of terminally differentiated epiphyseal chondrocytes are absent or expressed at negligible levels in articular chondroprogenitors, restricting their differentiation to the production of hyaline-like cartilage [27].

Defining the optimal pathways for chondrogenic differentiation and organized extracellular matrix production of tissue specific chondroprogenitors is therefore an important objective for tissue engineering cartilage and developing effective cell therapies. While chondrogenesis of epiphyseal chondroblasts and MSCs has been extensively described, and shown to be induced in medium containing transforming growth factor beta-1 (TGFβ1), dexamethasone, ascorbate, and insulin [28], there have been no comparable studies to determine the optimal chondrogenic medium for articular chondroprogenitors. TGFβ stimulates chondrogenesis in articular progenitors, although cartilage production is principally confined to the periphery of high-density pellet cultures, indicating a lack of potency [26].

The unique developmental history of articular chondroprogenitors led us to hypothesize that there are more potent chondrogenic factors for this cell type. Therefore, to determine the optimal chondrogenic medium for articular chondroprogenitors, we screened cells against a panel of known chondrogenic factors using high-density pellet culture as a means to analyze their potency.

## Materials and Methods

### Chondroprogenitor isolation

Articular cartilage tissue was harvested from freshly obtained healthy bovine metacarpophalangeal (MCP) joints (Cig Calon Cymru). Swansea University is registered for use of animal by-products as required under the requirements of Article 23 (EC) No, 1069/2009, and the work carried out in this study using these products was following institutional approval. Cartilage was removed from MCP joints of immature 7-day-old steers under sterile conditions and washed in Dulbecco's modified Eagle's medium (DMEM; Gibco) before sequential digestion with pronase (Roche) at 70 U/mL (0.2% w/v) in DMEM for 2 h, with the solution decanted and then medium added containing DMEM, 10 mM HEPES pH 7.4, 50 μg/mL gentamicin, 1% fetal bovine serum (FBS) with collagenase from *Clostridium histolyticum* (Sigma) at 300 CDU/mL (0.04% w/v) for 16 h, using a tube rotator or roller (Miltenyi Biotec) at 37°C and 5% CO_2_. Tissue digests were passed through a gravity driven nylon 40 μm cell strainer (Corning) to generate a single cell suspension.

Chondroprogenitor isolation was performed by differential adhesion of chondrocytes to plastic six-well plates (Greiner) that were precoated with 10 μg/mL of fibronectin (0.1% solution from bovine plasma; Sigma) in phosphate-buffered saline (PBS, pH 7.4) with 1 mM MgCl_2_ and 1 mM CaCl_2_ for 24 h at 4°C. Approximately 1,000 cells per well in 1.5 mL DMEM were incubated for 20 min on the fibronectin-coated plates at 37°C in a CO_2_ incubator, after which, nonadherent cells were removed and 3 mL of standard culture medium, DMEM (1 g/L glucose), 50 μg/mL ascorbic acid-2-phosphate, 10 mM HEPES pH 7.4, 1 mM sodium pyruvate, 2 mM l-glutamine, and 10% FBS and 50 μg/mL gentamicin added to each well. After 6 days of culture, well-spaced cell colonies of more than 32 cells, therefore excluding transit-amplifying cells, were isolated using sterile cloning rings (Sigma) using trypsin/ethylenediaminetetraacetic acid (EDTA) and transferred to six-well plates for culture expansion in standard culture medium. Unexpanded freshly isolated full-depth chondrocytes used for differentiation assays using the same basal chondrogenic medium as described below were from the same source and used following tissue digestion and cell straining.

### Chondroprogenitor differentiation

Basal medium for chondrogenic differentiation was composed of DMEM/F12 nutrient mix (1:1 with GlutaMAX, 17.25 μg/L l-proline, 3.151 g/L glucose; Cat. No. 31331-028; Gibco), supplemented with 10% heat-inactivated (60°C for 45 min) FBS, 100 μg/mL L-ascorbic acid 2-phosphate, 1% insulin-transferrin-selenium (ITS-X; Thermo Fisher Scientific), 10 mM HEPES pH 7.4, and 50 μg/mL gentamicin. Chondrogenic factors used in this study are listed with the final concentration used in pellet culture shown in brackets; chelerythrine chloride (CCl), a cell-permeable inhibitor of protein kinase C (0.66 μM), dibutyryl-cAMP (db-cAMP) a cell-permeable cyclic AMP analog that activates cAMP-dependent protein kinases (0.5 mM; Bio-Techne Ltd.), concanavalin A from *Canavalia ensiformis* (3 μg/mL), C-natriuretic peptide (CNP; 0.1 μM), ethanol (1.5% v/v; all Sigma-Aldrich), TGFβ1/2/3 (10 ng/mL), and bone morphogenetic protein (BMP) 2/9 (100 ng/mL; all PeproTech EC, Ltd.). For three-dimensional pellet culture, individual chondroprogenitor clones between 22 and 27 population doublings cells were trypsinized and 5 × 10^5^ cells were added to a sterile Eppendorf tubes in 1000 μL basal chondrogenic medium. The cell suspension was then centrifuged at 315 *g* for 5 min at room temperature to enable pellet formation, then incubated at 37°C and 5% CO_2_. After 24 h, cell pellets were gently aspirated with surrounding medium from the Eppendorf surface using a pipette to facilitate pellet rounding. Pellets were incubated with fresh medium every 72 h until the end of the culture period [29]. For differentiation on two-dimensional plastic, individual chondroprogenitor lines were seeded onto six-well dishes at a concentration of 1 × 10^5^ cells per well in standard culture medium. Each culture plate was then incubated at 37°C and 5% CO_2_ until the well was 80% confluent, upon which the medium was aspirated and 3 mL of prewarmed chondrogenic medium with or without growth factor added. The plate was then incubated at 37°C and 5% CO_2_ and medium changed once until analysis at 4 days posttreatment.

### RNA extraction

#### Pellets

Stored frozen pellets were thawed and lysis buffer RLT added (RNAEasy kit; Qiagen). Pellets were then mechanically homogenized for 30 s using a TissueRuptor device (Qiagen) using sterile probes. Total RNA was extracted using RNeasy columns with a DNase1 on-column digest as per manufacturer's instructions.

#### Reverse transcription-quantitative polymerase chain reaction

Complementary DNA (cDNA) was synthesized using ∼100 ng total RNA using standard methods. Quantitative polymerase chain reaction (qPCR) was performed using a Bio-Rad CFX96 thermal cycler using 25 μL reaction volumes in 96-well plates (Bio-Rad). Each reaction contained 3.5 mM MgCl_2_, 200 μM dNTPs, 0.3 μM forward and reverse primers, 0.025 U/μL Taq polymerase, and SYBR Green (GoTaq qPCR Master mix; Promega) with programmed reaction conditions of 1 cycle of 95°C for 10 min, 40 cycles of 95°C for 30 s, 55°C for 30 s, 72°C for 1 min, and 1 cycle of 72°C for 5 min and then 4°C. Absolute values for gene expression were calculated from standard curves generated using cloned and sequence-verified, serially diluted, plasmid template DNA. Primer sequences used for reverse transcription-qPCR) have been previously published [30–32].

### Biochemical compositional analysis

Before biochemical analyses, frozen cell pellets were thawed and solubilized by incubation in papain digestion buffer (20 mM NaAc pH 6.8, 1 mM EDTA pH 8.0, 2 mM dithiotheitol, and 300 μg/mL papain from *Papaya latex*; Sigma) at 60°C for 1 h. The Quant-iT PicoGreen dsDNA Assay Kit (Thermo Fisher Scientific) was used to measure DNA content using 50 μL volume of papain digested pellet according to the manufacturer's protocol and measured using a FLUOstar Omega plate reader (BMG Labtech). Data were compared to a linear standard curve (0–20 μg/mL) made using calf thymus DNA. To calculate cell number, DNA values were divided by 7.7 pg, the approximate weight of the bovine genome [33]. For sulfated glycosaminoglycan (sGAG) measurements, 20 μL of papain digested pellet sample was added to 200 μL of DMMB reagent (16 mg/L dimethylmethylene blue, 3 g polyvinyl alcohol 30–70 kDa, 3.04 g glycine, 2.37 g NaCl, and 95 mL 0.1 M HCl) in a 96-well plate before shaking for 5 s. The concentration of sGAG in each sample was determined by spectrophotometric measurement of absorbance at 525 nm and compared against a standard curve of shark chondroitin-4-sulphate (0–40 μg/mL; Sigma). Collagen content in pellets was determined using an assay to measure hydroxyproline in papain digested samples and comparing values against a standard curve of hydroxyproline (0–100 μg/mL).

### Histological analysis

Cell pellets were washed with PBS then fixed in 10% neutral buffered formyl saline (NBFS) for 12 h at 4°C, then processed for wax embedding. sGAG deposition was observed by using the metachromatic dye toluidine blue at 1% aqueous concentration for 60 s. To visualize collagen content and fibril alignment, rehydrated sections were stained in 0.1% w/v picrosirius red in saturated picric acid for 30 min.

### Immunohistochemistry

Formalin-fixed paraffin-embedded samples were sectioned to a thickness of 5 μm before immunohistochemical labeling. Sections were dewaxed in Histo-Clear (National Diagnostics) and rehydrated through an ethanol gradient before being brought to deionized water. Antigen retrieval was performed in two stages; an overnight incubation at 65°C in a pH 9 10 mM Tris, 1 mM EDTA, 0.05% Tween-20 buffer solution, followed by incubation with 1 mg/mL hyaluronidase (bovine testes; Sigma) in phosphate-buffered saline (PBS) buffer at 37°C for 1 h. Sections were blocked using horse serum (2.5%; Vector Laboratories) for 30 min. Labeling was performed against aggrecan [1-C-6, Developmental Studies Hybridoma Bank (DSHB)], collagen type I (C2456; Sigma), and collagen type II (II6B3, DSHB). Primary antibodies were diluted 1:10 for aggrecan and collagen type II and 1:2,000 for collagen type I, in PBS Tween-20 (0.05%). Primary antibody detection was performed using the RTU biotinylated pan-specific antibody, RTU streptavidin/peroxidase complex and ImmPACT NovaRED Peroxidase Substrate (Vector Laboratories), according to manufacturers' instructions. Sections were counterstained with Meyer's hematoxylin (TCS Biosciences) and mounted using DPX Mounting Medium (Electron Microscopy Sciences).

### Atomic force microscopy

Chondroprogenitor cell monolayer cultures were imaged and analyzed using quantitative nanomechanical mapping (QNM) and force volume (FV) to assess changes in morphological and nanomechanical phenotype between the different populations at expansion stage and under differentiation conditions. Imaging experiments were performed using a Bioscope Catalyst (Bruker) instrument in either QNM or FV mode. High aspect ratio silicon probes, MLCT-D (Bruker), were used for the experiments with a spring constant of 0.03 N/m and a cantilever calibrated using in house manufacturer protocols before each experiment. Cells were imaged alive in DMEM without phenol red (Gibco) media, at 37°C. Care was taken to avoid the generation of imaging artefacts throughout. At least 6 cells per sample were analyzed taking 25 FV curves per cell in a 2.5 μm^2^ area in 30 min not to lose the phenotype or adherence of the cells. A 1 μm ramp size was applied to the sample with a force of 500 pN, the ramp rate was 1.03 Hz with constant forward and reverse tip velocity of 2.06 μm/s. When imaging in QNM mode, peak force amplitude and frequency were set at 500 nm and 0.25 kHz, respectively. The scan rate was 0.5 Hz and the 5.05 μm/s tip velocity, applying a force between 1 and 0.5 nN with feedback gain of 1.000.

### Statistical analyses

All statistical approaches were performed using SPSS software version 19.0 for the windows operating system. All data sets were first analyzed for normality using the Anderson Darling test. Statistical significance was calculated using one-way analysis of variance. Post hoc analysis was performed using Tukey's honestly significant difference method. All statistical significance threshold values are *P* < 0.05, unless otherwise stated in the text.

## Results

**FIG. 1.**
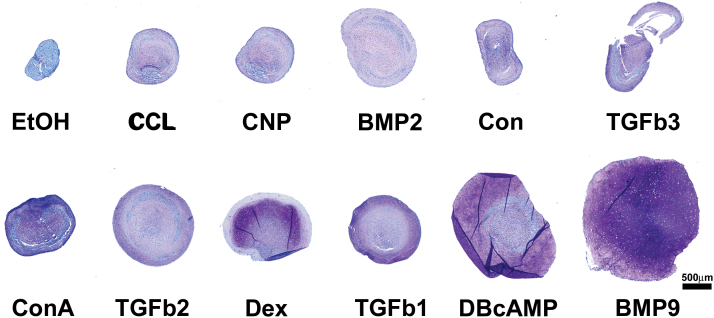
Toluidine blue-stained sections of chondroprogenitor pellets grown as high-density pellet cultures. Pellets of 5 × 10^5^ cells were grown in minimal chondrogenic medium (Con) or supplemented with EtOH, CCl, ConA, CNP, BMP2/9, TGFβ1–3, dexamethasone (Dex), and dc-AMP. Pellets are ordered according to the quantity of sGAG normalized to DNA content (Fig. 2A). Representative images of three independent experiments. Scale bar equals 500 μm. BMP, bone morphogenetic protein; CCl, chelerythrine chloride; CNP, C-natriuretic peptide; ConA, concavalin-A; dc-AMP, dibutyryl cyclic adenosine monophosphate; EtOH, ethanol; sGAG, sulfated glycosaminoglycan; TGFβ, transforming growth factor beta. Color images are available online.

Articular cartilage progenitors from immature cartilage were cultured as high-density cell pellets for 21 days in the presence of minimal chondrogenic culture medium that was supplemented with previously characterized chondrogenic factors, TGFβ1–3 (10 ng/mL), BMP 2/9 (100 ng/mL), dexamethasone (Dex), CNP, db-cAMP, concavalin-A (ConA), ethanol (EtOH), and CCl at concentrations previously shown to induce differentiation in receptive mesenchymal cells [34–43]. Histologic examination of toluidine blue-stained sections of cartilage pellets showed an increasing gradation in size and staining intensity from ethanol to BMP9-treated chondroprogenitors (Fig. 1). Only BMP9-treated chondroprogenitors showed uniform deep purple toluidine blue staining throughout the pellet depth, with the other factors showing variegated staining of pellets due either to the lack of sulfated proteoglycan or to the metachromatic nature of the dye, where reduced staining is visualized as a blue color.

**FIG. 2.**
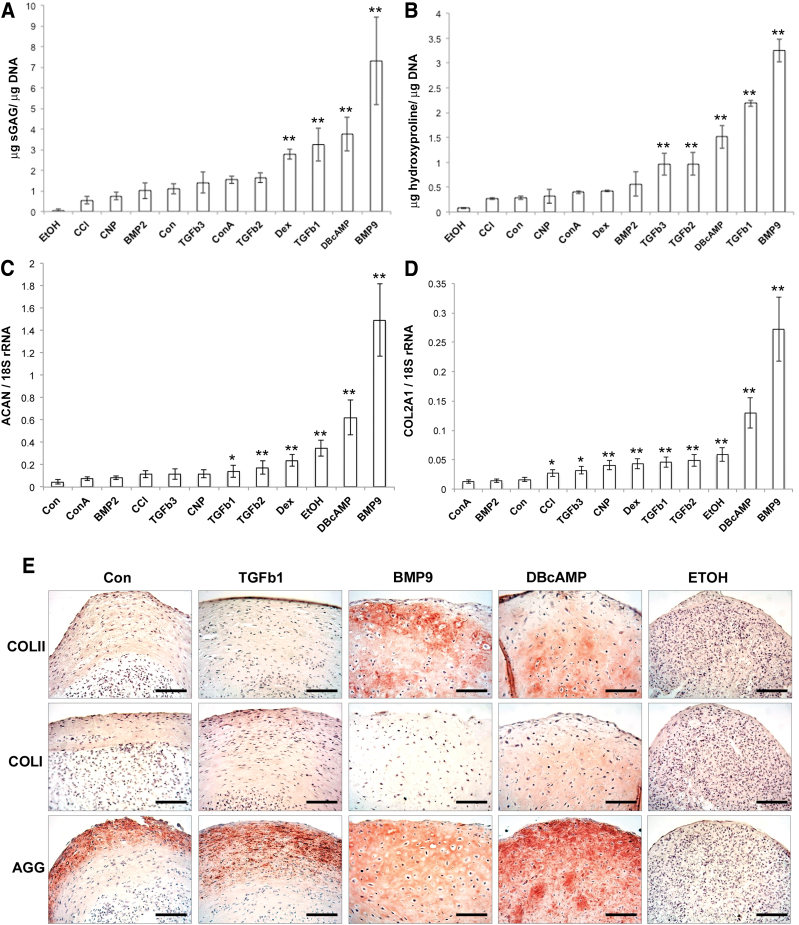
Quantification of **(A)** sGAG (*n* = 6), **(B)** hydroxyproline content (*n* = 6), and gene expression levels of **(C)** ACAN and **(D)** COL2A1 in chondroprogenitor pellet cultures (*n* = 4 for both genes). **(E)** Selected pellets were screened for reactivity against antibodies to collagen (Col) types I and II and aggrecan (Agg). Scale bar equals 100 μm. *P < 0.05, **P < 0.01 versus control. ACAN, aggrecan; COL2A1, collagen type II. Color images are available online.

Quantification of sGAG content using the DMMB assay normalized to DNA content revealed a 6-fold increase in sGAG in BMP9 pellets compared to minimal medium alone (*P* < 0.01) and 2.3-fold greater accumulation of sGAG than TGFβ1-treated pellets (*P* < 0.01) (Fig. 2A). Other than BMP9, only dexamethasone 2.4-fold, TGFβ1 2.8-fold, and db-cAMP 3.3-fold showed significantly increased sGAG content (*P* < 0.01) compared to pellets cultured in minimal medium. When hydroxyproline content was measured in pellets, TGFβ2 3.4-fold, TGFβ3 3.4-fold, db-cAMP 5.8-fold, TGFβ1 7.8-fold, and BMP9 11.5-fold-treated pellets showed a greater deposition of collagen compared to pellets cultured in minimal medium (*P* < 0.01) (Fig. 2B). Analysis of aggrecan (ACAN) and collagen type II (COL2A1) gene expression in pellets led to broadly similar changes with BMP9 pellets showing 33-fold greater ACAN gene expression compared to pellets in minimal medium (*P* < 0.01) and 10.6-fold more than in TGFβ1 pellets (*P* < 0.01) (Fig. 2C). COL2A1 gene expression was similarly 17-fold higher in BMP9 compared to minimal medium pellets (*P* < 0.01) and 5.4-fold higher than TGFβ1 pellets (*P* < 0.01) (Fig. 2D). Of note, the greatest contrast between biochemical content and gene expression measurements were for ethanol-treated pellets, where gene expression values for ACAN and COL2A1 were among the highest, while the content values for proteoglycan (sGAG) and collagen (hydroxyproline) normalized to DNA were the lowest.

Immunohistochemical labeling of sections from pellets cultured in minimal chondrogenic medium, or supplemented with TGFβ1, BMP9, db-cAMP, or ethanol, against antibodies labeling collagen types I and II, and aggrecan illustrated not only the differences in relative amount of protein deposited in the extracellular matrix of pellets but also their differential localization (Fig. 2E). BMP9-treated pellets displayed labeling for collagen type II and aggrecan throughout the full depth of the pellet, whereas TGFβ1-treated pellets had higher collagen type I labeling throughout the pellet with aggrecan labeling concentrated at the pellet periphery as predicted from toluidine blue staining (Fig. 1).

**FIG. 3.**
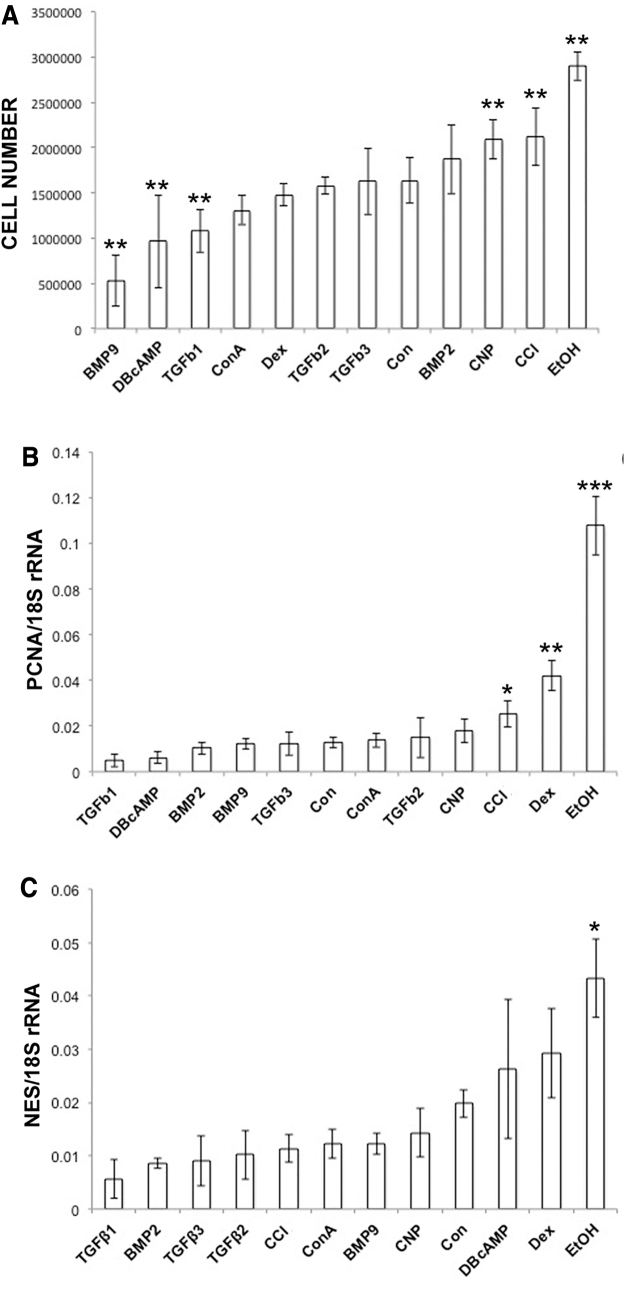
Cellular content of cultured chondroprogenitor pellets cultured in minimal chondrogenic medium, or the same medium supplemented with various chondrogenic factors. **(A)** Cell number (*n* = 6) was calculated by dividing total DNA content of papain digested pellets by the approximated weight of DNA in a single bovine chondrocyte (calculated as 7.7 pg [33]). Gene expression analysis using qPCR of **(B)** PCNA and **(C)** NES in chondroprogenitor pellets (*n* = 4 for both genes). *P < 0.05, **P < 0.01, ***P < 0.001 versus control. NES, nestin; PCNA, proliferating cell nuclear antigen; qPCR, quantitative polymerase chain reaction.

The DNA content of chondroprogenitor pellets, converted to cell number, had two interesting features; first, BMP9 pellets exhibited the smallest average increase in cell number from the starting count of 5 × 10^5^ cells (26%), this was 2.6-fold less than average cell counts for pellets cultured in minimal medium (*P* < 0.01). Second, CNP, CCl, and ethanol-treated pellets exhibited significant increases in cell number when compared to pellets cultured in minimal medium alone (*P* < 0.01) (Fig. 3A). Gene expression analysis using primers for proliferating cell nuclear antigen (PCNA) correlated with DNA measurements showing increased cell number most notably in ethanol-treated pellets (*P* < 0.01) (Fig. 3B). qPCR analysis also showed that nestin (NES), a marker for MSCs, was significantly increased in ethanol treated pellets only compared to pellets in minimal chondrogenic medium. Analysis of the relationship between DNA and proteoglycan content of pellets showed a strong negative correlation (*r* = −0.88, *P* < 0.001) between the two variables with sGAG content decreasing with increasing DNA content.

**FIG. 4.**
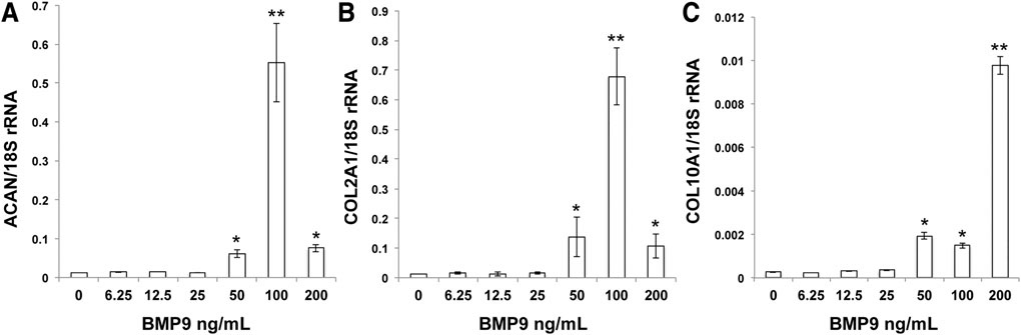
Determining the optimum BMP9 concentration for chondrogenic induction of immature articular chondroprogenitors. Progenitors (*n* = 4) grown in monolayer to near confluence (80%) were exposed to increasing concentration of BMP9 (0–200 ng/mL) for 4 days and then assayed for gene expression using qPCR of **(A)** ACAN, **(B)** COL2A1, and **(C)** COL10A1. *P < 0.05, **P < 0.01 versus control. COL10A1, collagen type X.

Titration of BMP9 growth factor to determine the optimum concentration for articular chondroprogenitor differentiation was examined in the range of 0–200 ng/mL of growth factor using gene expression analysis of ACAN, COL2A1, and collagen type X (COL10A1) of cells grown in monolayer (Fig. 4). These data show that 100 ng/mL BMP9 was the optimum concentration for chondrogenic induction of progenitors, ACAN and COL2A1 gene expression was highest at this concentration (*P* < 0.001), whereas COL10A1 gene expression was greatest using BMP9 at 200 ng/mL. While COL10A1 levels at 100 ng/mL BMP9 also induced a significant increase, they were 7-fold less than using the highest BMP9 concentration (1.4 × 10^−3^ ± 1 × 10^−4^ vs. 9.7 × 10^−3^ ± 4 × 10^−4^); in absolute terms, the expression level of COL10A1 was ∼450-fold less than COL2A1 gene expression levels (0.68 ± 0.01) at the same concentration of BMP9 (100 ng/mL).

**FIG. 5.**
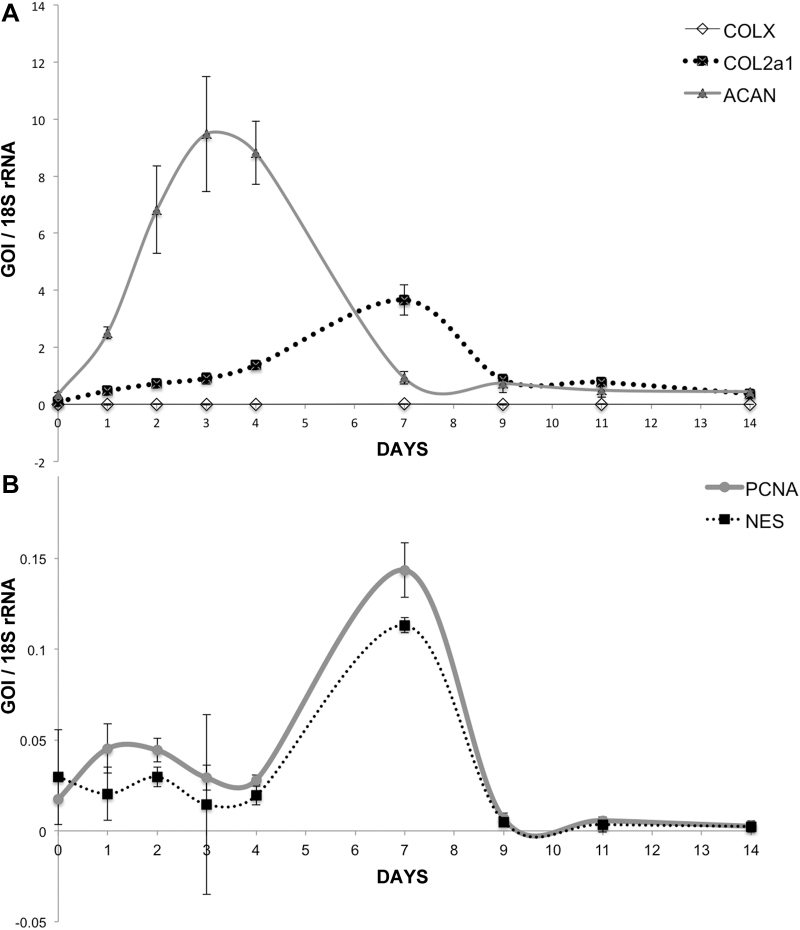
Quantitative analysis of gene expression in BMP9-treated chondroprogenitor pellets over 14 days. Chondrogenitor pellets (*n* = 3) from 9 time points over 14 days were processed to isolate RNA and assayed using RT-qPCR gene expression of **(A)** COL2A1, ACAN, and COL10A1 and **(B)** PCNA and NES. RT-qPCR, reverse transcription-quantitative polymerase chain reaction.

Developmental analysis of chondrogenesis has revealed that extracellular matrix accumulation occurs in a phasic manner. To examine if this was the case with BMP9-treated chondroprogenitors, we measured the change in gene expression of ACAN, COL2A1, and COL10A1 in chondroprogenitor pellets over a period of 14 days. Visual observation of pellet growth in BMP9 supplemented minimal medium indicated that there was rapid growth over the first 7–10 days beyond which point any increase in size was imperceptible (data not shown). Gene expression data collected over a 14-day period and normalized to pellets grown in minimal chondrogenic medium (Fig. 5A) showed that ACAN gene expression was maximal between days 2 and 4 and fell for the remaining period of analysis (day 0 0.35 ± 0.07, day 3 9.48 ± 2.02, and day 7 0.93 ± 0.22). Similarly, COL2A1 gene expression peaked between days 6 and 8 (day 0 0.09 ± 0.008, day 7 3.66 ± 0.53, day 9 0.87 ± 0.15). The levels of COL10A1 gene transcription did not rise significantly over 14 days and were maximal at day 7 (0.017 ± 0.016) ∼215-fold less in absolute terms compared to COL2A1 at day 7. We also examined markers of cellular proliferation, PCNA, and MSC marker, NES, over the same time period (Fig. 5B). We noted that the pattern of gene expression, which was maximal between days 6 and 8, was similar for both PCNA and NES.

**FIG. 6.**
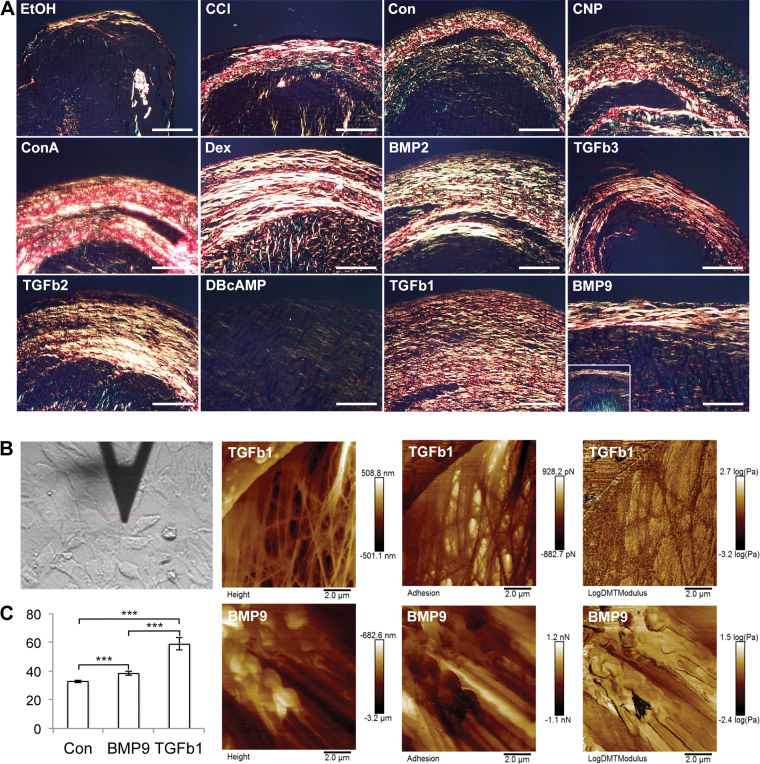
Organization of collagen fibrils in differentiated chondroprogenitor pellets using polarized light microscopy. **(A)** Pellets grown for 21 days were sectioned and stained with 1% PSR, washed, and mounted using DPX under coverslip. Scale bar equals 100 μm. **(B)** Atomic force microscopy analysis of the extracellular matrix deposited by chondroprogenitors cultured in monolayer undergoing differentiation following exposure to TGFβ1 and BMP9. Representative images show the fibrillar nature of the matrix produced by TGFβ1-treated progenitors compared to BMP9 cells. **(C)** Stiffness of the extracellular matrix surrounding chondroprogenitors grown in minimal chondrogenic medium or supplemented with TGFβ1 or BMP9 (*n* = 4, ***P < 0.001 vs. control). Representative images from two independent experiments. PSR, picrosirius red. Color images are available online.

**FIG. 7.**
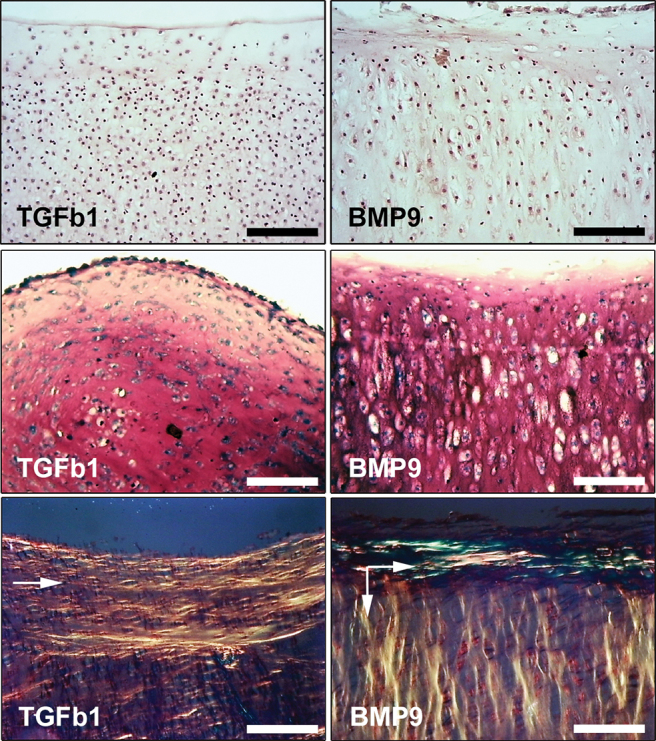
Organization of collagen fibrils in pellet cultures of freshly isolated full depth immature chondrocytes. Pellets composed of 1 × 10^5^ cells each were cultured in standard chondrogenic medium for 21 days with either TGFβ1 (10 ng/mL) or BMP9 (100 ng/mL). Pellets were processed to wax, sectioned, and stained with hemotoxylin and eosin (*upper panel*), safranin-O (*middle panel*), and PSR (*lower panel*). Scale bar equals 100 μm. PSR-stained sections were visualized using polarized light microscopy and the bulk orientation of collagen fibrils is shown by the *arrows* in each image (*lower panels*). Representative images from three independent experiments. Color images are available online.

Collagen deposition is a critical factor in the extracellular matrix of cartilage tissue, and the manner in which the collagen fibrils are aligned in the tissue affects their overall contribution to the structure and function of tissue. Using picrosirius red staining and polarized light microscopy (PLM), we observed patterns of birefringence characteristic of collagen fibril alignment in pellet cultures (Fig. 6A). The most striking feature was the parallel alignment of thick fibrils within pellets most notable in TGFβ1-treated chondroprogenitors. At lower magnification, some evidence of anisotropic collagen fibril organization could be observed in pellets cultured with CCl, dexamethasone and BMP9. To confirm the differential nature of the extracellular matrix induced by different growth factors, atomic force microscopy (AFM) analysis was to determine the biophysical characteristics of matrix deposited surrounding cells after TGFβ1 or BMP9 exposure in monolayer culture. These experiments show that cell stiffness increases significantly upon differentiation with both growth factors, but the magnitude of increase is greater using TGFβ1 (Fig. 7A). Topographic maps of the matrix surrounding cells showed more fibrous matrix in TGFβ1-treated chondroprogenitors, a feature much less prominent in matrix surrounding BMP9-treated cells (Fig. 6B).

We reasoned that predifferentiated cells, that is, full-depth chondrocytes isolated from intact immature cartilage, and not requiring chondrogenesis before producing extracellular matrix, would allow us to distinguish the different functions of BMP9 on chondrocytes and cartilage. PLM analysis of 21-day pellet cultures of 5 × 10^5^ chondrocytes from immature cartilage treated with either TGFβ1 or BMP9 revealed profound differences in collagen and cellular organization (Fig. 7). TGFβ1-treated pellets displayed characteristic parallel arrays of collagen alignment that extended from the periphery deep into the pellet, whereas BMP9-treated pellets displayed an anisotropic organization typically found in mature cartilage. Columnar arrays of collagen fibrils perpendicular to the surface zone were present in the deeper zones of BMP9-treated pellets and chondrocytes were aligned in the direction of these fibrils (arrowed in Fig. 7).

## Discussion

The results of this study demonstrate that BMP9 is a potent chondrogenic factor for immature articular cartilage-derived chondroprogenitors. PLM also reveals that BMP9 is capable of inducing morphological changes either by stimulating deposition or reconfiguration of extracellular matrices to produce an anisotropic structure that mirrors that seen during postnatal maturation [30,44]. This study was motivated by observations that articular cartilage-derived chondroprogenitors are incompletely differentiated in pellet culture when stimulated with TGFβ1 [26]. In selecting candidates for screening progenitors for enhanced chondrogenesis, we examined previous literature identifying chondrogenic factors for embryonic chick limb mesenchyme. Our reasoning for this approach was that chondroprogenitors used in this study were derived from newborn bovine cartilage whose structural organization is isotropic [23,24], therefore, it was likely that they would be more receptive to factors known to act on immature mesenchymal progenitors. Of particular note, we chose to include BMP9 (also known as growth and differentiation factor-2, GDF2) based on studies showing that it could induce chondrogenesis and phylogenetic data classifying it and BMP10 into a separate group from other BMPs [45]. The concentrations of chondrogenic factors used in this study were based on previously published data using identical or similar cells, an inherent limitation of this approach therefore is the possibility that some factors may not be present at their optimal concentration.

Following pellet culture for 21 days with candidate chondrogenic factors, BMP9 was the most potent as viewed by toluidine blue staining of sectioned tissue. Biochemical and gene expression analyses confirmed histological observations of BMP9 enhancing differentiation of chondroprogenitors especially when compared to growth factors TGFβ1–3. Of particular note was immunohistochemical data that showed the distribution of proteoglycan aggrecan, and collagen type II antibody labeling was evenly spread in BMP9 and db-cAMP compared to TGFβ1-treated pellets. We also observed a strong negative association between cellular proliferation and sGAG deposition, where BMP9-treated pellets did not show appreciable increase in cell number from initial seeding. Without monitoring cell death or proliferation directly, it is not possible to eliminate the possibility that there was a balance between cell death and proliferation within BMP9 pellets.

Analysis of gene expression of PCNA in BMP9-treated pellets over a period of 14 days showed that there was a peak in expression at day 7 and this coincided with a peak in expression of nestin transcripts. Nestin is a class VI intermediate filament protein that forms complex heterodimers and heterotetramers, and there is strong evidence in MSCs implicating it as a marker for multilineage progenitor cells [46]. Nestin expression identifies MSC subpopulations essential for hematopoietic stem cell expansion in the bone marrow niche [47], and a study by Kang et al. examining the role of IL6 in MSC cartilage differentiation showed that nestin antibody labeling colocalizes with MSC marker CD166 in macroscopically normal cartilage [48]. Therefore, based on these indirect observations, we predict that a subpopulation of chondroprogenitors are maintained within the growing cartilage pellet and may act as a reserve progenitor pool to stimulate further growth or repair [22,49,50].

Several reasons may explain why BMP9 is more effective than the other factors used in this study. Pulse-chase experiments using ^35^S-sulfate and ^3^H-proline by Hills et al. showed that intact juvenile (1- to 3-month-old) bovine cartilage explants treated with BMP9 increase their synthesis of proteoglycan and collagen extracellular matrix components and simultaneously exhibit reduced turnover of labeled proteins [51]. Decreased proteolysis of extracellular matrix mediated by BMP9 could be accomplished through suppression of protease activity by transcriptional mechanisms or indirectly through upregulation of tissue inhibitor of metalloproteinase gene transcription [52,53]. Conversely, the addition BMP9 (and BMP2) can overcome the inhibitory effect of physiologically relevant levels of proinflammatory cytokine IL1β on bone marrow-derived MSC chondrogenesis by maintaining gene expression of COL2A1, ACAN, and SOX9 [39].

BMP2 preferentially binds to activin receptor-like kinase-3 and -4 (ALK3/4) receptors and this probably accounts for the differential induction of chondrogenesis in progenitors when compared to BMP9, as BMP9 ligands signal through ALK1/2 receptors as shown by inhibition of in vitro osteogenesis and in vivo ectopic bone formation following transfection of dominant-negative ALK receptors into bone-derived MSCs [54,55]. There is also evidence to suggest that BMP signaling predominates in the earliest stages of differentiation. Dedifferentiated articular chondrocytes cultured as pellets initially display higher BMP4 expression, SMAD 1/5/8 phosphorylation, and SOX9 protein levels and undergo faster redifferentiation when compared to bone marrow-derived MSCs, highlighting the early role of BMPs in chondrogenesis [56]. Furthermore, transgenic studies conclude that TGFβ growth factors are not essential for prechondrogenic condensation formation; conditional ablation of ALK5 using Dermo-Cre targeting mesenchymal progenitors does not affect either condensation or cartilage formation [57], indicating that stimulation of chondrogenesis through activation of SMAD 1/5/8 predominates in the early stages of differentiation.

During postnatal growth and homeostasis, TGFβ signaling through ALK5 receptors act as an inhibitory stimulus on chondrocyte terminal differentiation and the adoption of a mineralizing phenotype in cells [58]. An age-associated imbalance in the ratio of ALK5 to ALK1 seen in osteoarthritic cartilage is also further evidence for the developmental and pathological context-dependent activities of BMP and TGFβ growth factors on chondrocytes [59]. Therefore, articular chondroprogenitors may be primed to undergo rapid chondrogenic differentiation following receptor-mediated SMAD 1/5/8 signaling and, in later stages, postcondensation, TGFβ-dependent SMAD 2/3 signaling may predominate to promote homeostatic growth and prevent mineralization in permanent chondrocytes.

PLM of cartilage pellets showed that for the most part, collagen fibrils were organized in highly fibrillar, annular, and concentric patterns. The exception were db-cAMP-treated pellets, where very weak birefringence signal was detected, indicating an unusual lack of fibril organization. In BMP9-treated pellets, there was evidence of fibrils organized perpendicular to the surface, but the birefringence signal was relatively weak (Fig. 6A, BMP9 inset). We hypothesized that the incomplete morphogenic effects of BMP9 on chondroprogenitors were due to the need for these cells first to undergo differentiation. Therefore, we cultured freshly isolated full-depth chondrocytes from immature cartilage with TGFβ1 and BMP9 for 21 days. BMP9-treated chondrocytes produced a birefringence pattern of collagen fibril alignment and organization indistinguishable from the pattern found in mature adult cartilage, with a thin ring of parallel fibers running across the peripheral boundary of the pellet below, which were fibers in antiparallel orientation. Chondrocytes in columnar organization and with enlarged chondrons were clearly visible in safranin O-stained sections of BMP9-treated pellets. In contrast, TGFβ1-treated chondrocyte pellets produced a broad fibrillar ring of collagen orientated parallel to the surface. AFM analysis of chondroprogenitors differentiated with TGFβ1 or BMP9 showed that TGFβ1-induced matrix deposition was predominantly fibrillar and significantly stiffer compared to BMP9-treated cells, which themselves produced a less fibrillar and more compliant matrix. Substrate compliance is known to alter not only cell phenotype but also plays a role in tissue development, where stiffer artificial matrices negatively regulate tissue architecture and differentiation [60].

We have previously proposed that a transient genetic program is activated to initiate postnatal maturation of articular cartilage [30–32], although it remains to be seen whether elements of this program have been invoked by BMP9 treatment. Also, in this study, we used progenitors and chondrocytes from immature cartilage and whether progenitors and chondrocytes from older structurally mature cartilage are similarly receptive to BMP9-induced chondrogenesis or morphogenic cues also remains to be tested. Understanding how articular cartilage undergoes the postnatal transition from isotropically organized immature cartilage to anisotropically mature cartilage is critical for tissue engineering, regeneration, and repair strategies. Whether chondroprogenitor proliferation at the joint surface initiates a process of appositional growth generating trailing pillars or stacks of daughter cells [24,61] or cellular hypertrophy, matrix deposition, and reorganization of chondrocytes underlies the growth and maturation of the tissue [62] are issues that need to be resolved. Our finding showing for the first time that postnatal maturation can be rapidly recapitulated in vitro may help to shed light on these competing hypotheses.

In conclusion, BMP9 is a potent chondrogenic factor for immature chondroprogenitors and additionally provides morphogenic cues for progenitors and differentiated chondrocytes to generate adult-like anisotropic organization. The identification of BMP9 opens up the possibility of using allogeneic culture-expanded progenitors for in vitro production of fully differentiated and functional implants for repair of localized cartilage lesions.
